# Obesity-driven extracellular vesicle signaling in cancer: mechanistic insights and clinical implications

**DOI:** 10.1038/s41389-026-00640-2

**Published:** 2026-07-01

**Authors:** Lakshmi Narasimhan Chakrapani, Jessica Velasquez, Joshika Suresh, Subasri Durga Sridhar, Ramya Sankaran, Suriya Prakash Arivazhagan, Archana Shanmugam, Kalpana Deepa Priya Dorayappan, David M. O’Malley, Thangavel Muthusamy, Karuppaiyah Selvendiran

**Affiliations:** 1 Division of Gynecologic Oncology, Department of Obstetrics and Gynecology, The James Comprehensive Cancer Center, The Ohio State University Wexner Medical Center, Columbus, OH USA; 2 Cellular and Molecular Biochemistry unit, Research and Development, Sree Balaji Medical College and Hospital, Bharath Institute of Higher Education and Research (BIHER), Chennai, Tamil Nadu India

**Keywords:** Cancer, Cell signalling

## Abstract

Obesity is a rapidly escalating global health challenge and a major, modifiable driver of cancer risk and poor outcomes across at least 13 malignancies, including endometrial, colorectal, breast, pancreatic, hepatic, renal, ovarian, and esophageal adenocarcinoma. Beyond endocrine and metabolic effects, obesity establishes a tumor-permissive systemic state characterized by adipose hypoxia, chronic inflammation, insulin resistance, and adipokine imbalance. Here, we synthesize evidence that extracellular vesicles (EVs) serve as a central mechanistic conduit through which these obesity-associated stress programs are transmitted to tumor and stromal compartments, driving cancer initiation, progression, immune evasion, metastasis, and therapy resistance. Obesity amplifies EV biogenesis and reprograms EV cargo through hypoxia- and inflammation-responsive signaling and altered endosomal trafficking, enriching EVs with coordinated lipid, RNA, and protein modules that durably rewire recipient cells. Functionally, obesity-conditioned EVs reinforce oncogenic growth and survival signaling, promote epithelial plasticity and angiogenesis, remodel the tumor microenvironment toward immune suppression and extracellular matrix reorganization, and facilitate pre-metastatic niche formation. Clinically, EVs provide a stable, information-rich substrate for liquid biopsy development, with emerging EV signatures showing promise for early detection, risk stratification, and longitudinal disease monitoring in obesity-associated cancers. We conclude by outlining key mechanistic, technological, and translational priorities required to advance EV-based biomarkers and to therapeutically disrupt obesity-driven intercellular communication.

## Introduction

The worldwide rise in obesity has been accompanied by a parallel increase in cancers that are strongly influenced by metabolic health, positioning excess adiposity among the most actionable determinants of cancer risk at the population level [1, 2]. Epidemiologic and experimental evidence now firmly establishes obesity as a biological condition that enhances cancer initiation, accelerates progression, and diminishes therapeutic responsiveness, thereby amplifying cancer-related morbidity and mortality across multiple tumor types [3–5].

Mechanistically, obesity induces a chronic, systemic state of metabolic and inflammatory stress characterized by insulin resistance, persistent low-grade inflammation, adipokine dysregulation, and hypoxia within expanding adipose depots [5–7]. These perturbations reshape endocrine signaling and immune homeostasis while creating metabolic conditions that favor malignant transformation, tumor progression and resistance [8–10]. However, the pathways by which obesity-derived signals are coordinated, stabilized, and transmitted across tissues to influence tumor ecosystems remain incompletely resolved.

Extracellular vesicles (EVs) have emerged as critical mediators of long-range intercellular communication capable of integrating and propagating complex biological information [11–13]. EVs are membrane-enclosed nanoparticles released by adipocytes, immune cells, stromal cells, endothelial cells, and tumor cells that transport bioactive lipids, nucleic acids, metabolites, and proteins to local and distant recipient cells [13–16]. In obesity, EV output increases substantially, and EV cargo composition shifts toward inflammatory, metabolic, and stress-responsive programs, enabling durable signal transfer that extends beyond the transient actions of soluble cytokines and adipokines [17–19].

A central challenge in the obesity–cancer field is that multiple mediators, cytokines, adipokines, metabolites, and immune-cell remodeling operate simultaneously, making it difficult to identify dominant causal conduits and clinically tractable biomarkers [20–22]. In this review, we propose an integrative framework in which EVs function as systems-level signal integrators and amplifiers, converting local adipose dysfunction into coordinated inter-organ communication that reshapes tumor cell behavior and the tumor microenvironment. Rather than cataloging individual EV cargos, we synthesize how obesity-associated hypoxia, inflammation, and metabolic stress converge on EV biogenesis, cargo loading, and trafficking to drive cancer-relevant phenotypes. We further highlight translational opportunities and technical priorities required to leverage EV biology for biomarker development and therapeutic intervention in obesity-associated cancers.

## A mechanistic spine for obesity-driven EV signaling in cancer

**Fig. 1:**
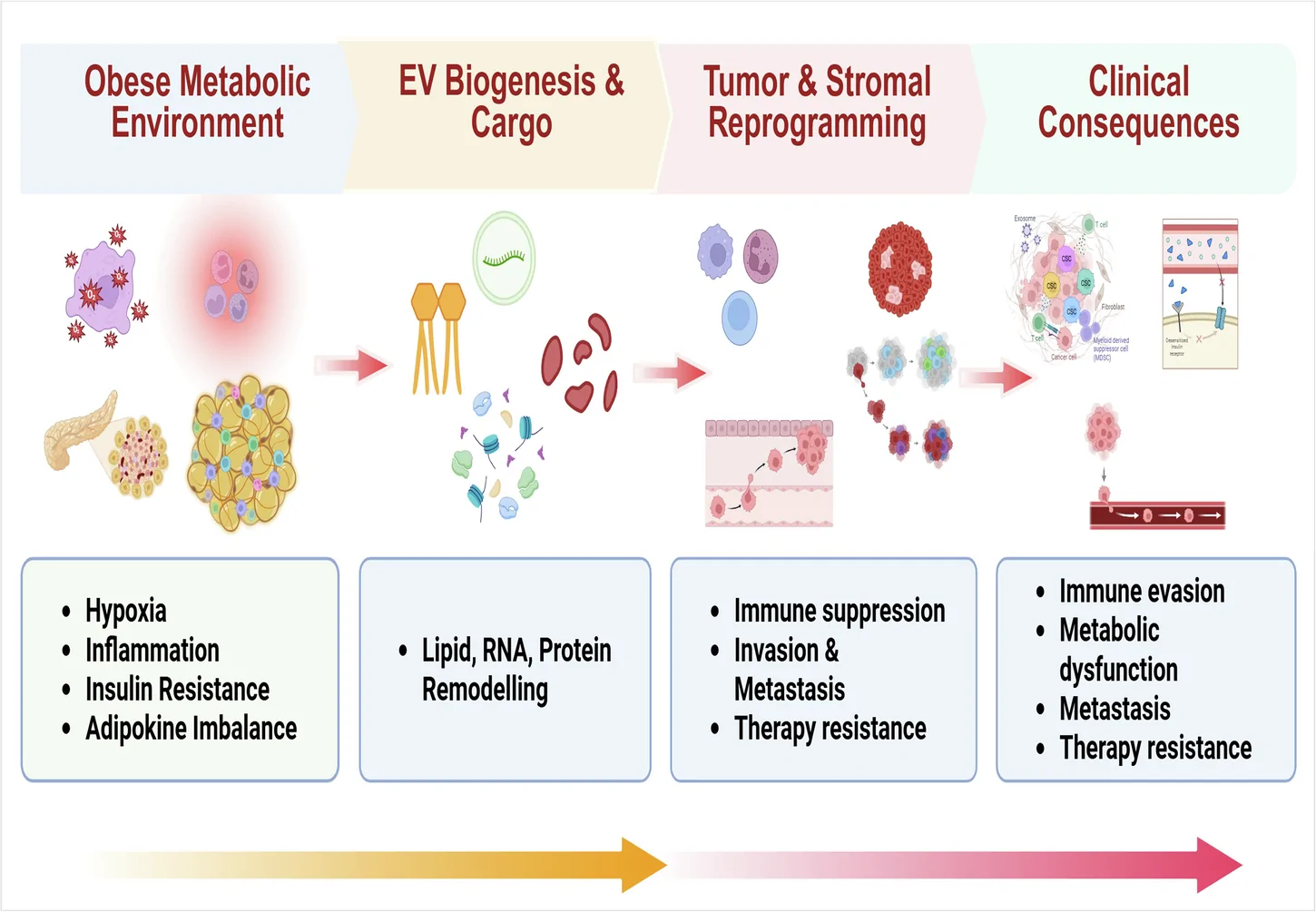
Mechanistic framework linking obesity, EV signaling, and cancer progression. Obesity-associated metabolic stress (hypoxia, inflammation, insulin resistance, adipokine imbalance) reprograms EV biogenesis and cargo, increasing release of lipid-, RNA-, and protein-enriched EVs. These EVs drive tumor and stromal remodeling, promoting immune suppression, metastasis, and therapy resistance, with implications for biomarker and therapeutic development.

To address the complexity of obesity-associated cancer biology, we organize EV-mediated signaling into a unified mechanistic spine consisting of four interconnected levels: (i) obesity-associated systemic stressors, (ii) EV biogenesis and cargo reprogramming, (iii) EV-driven cellular and microenvironmental outputs, and (iv) translational consequences (Fig. 1).

### (i) Obesity-associated systemic stressors

Obesity imposes a persistent physiologic stress state marked by adipose tissue hypoxia, chronic inflammation, lipid overload, insulin resistance, and adipokine imbalance [23–25]. These conditions activate conserved stress-response pathways, including hypoxia-inducible and inflammatory transcriptional programs, that rewire cellular trafficking, metabolism, and secretion [26, 27]. Importantly, these stressors do not act in isolation but converge to reshape intercellular communication at the systems level.

### (ii) Reprogramming of EV biogenesis and cargo

In obese states, hypoxia- and inflammation-responsive signaling alters endosomal trafficking, multivesicular body fate, and vesicle release dynamics, leading to increased EV output across adipose, immune, stromal, and tumor compartments [28, 29]. Concurrently, cargo loading is selectively reprogrammed, favoring the incorporation of bioactive lipids, regulatory RNAs, and stress- or oncogenic signaling proteins [16, 30]. This coordinated remodeling enables EVs to encode integrated metabolic–inflammatory programs rather than isolated molecular signals.

### (iii) EV-driven outputs in tumor and stromal compartments

Upon uptake by recipient cells, obesity-conditioned EVs activate growth and survival signaling, promote epithelial plasticity and metabolic adaptation, suppress anti-tumor immunity, and remodel stromal and extracellular matrix architecture [31, 32]. Within the tumor microenvironment, these effects manifest as enhanced angiogenesis, immune evasion, invasion, and resistance to therapy [33]. At distant sites, EVs contribute to pre-metastatic niche formation by reprogramming stromal, immune, and endothelial cells toward tumor-permissive states [32, 34].

### (iv) Translational consequences

Because EVs encapsulate coordinated molecular programs reflective of their cells of origin, they provide a mechanistically grounded substrate for biomarker development [35, 36]. At the same time, their biogenesis, cargo loading, and uptake pathways represent actionable intervention points through which obesity-driven tumor–host communication may be therapeutically disrupted.

## Obesity, hypoxia, and reprogramming of extracellular vesicle biogenesis

A defining feature of obesity is chronic hypoxia within expanding adipose depots, arising from increased tissue mass, impaired vascularization, and elevated metabolic demand [37]. Adipose hypoxia functions as a master upstream stressor that integrates inflammatory, metabolic, and trafficking pathways, thereby reshaping EV biogenesis across adipocytes, immune cells, stromal cells, and tumor cells [13, 38].

Hypoxia-inducible factor–1α (HIF-1α) and signal transducer and activator of transcription–3 (STAT3) represent central nodes linking obese tissue stress to EV production. In hypoxic adipose and tumor microenvironments, reciprocal activation of HIF-1α and phosphorylated STAT3 establishes a feed-forward transcriptional program that promotes inflammatory signaling, metabolic adaptation, and vesicular trafficking [39–41]. STAT3 activation, driven by obesity-associated cytokines such as IL-6 and receptor tyrosine kinase signaling, induces transcriptional programs that regulate endosomal sorting machinery, while HIF-1α stabilization promotes metabolic rewiring and suppresses lysosomal degradation pathways [13, 39]. Together, these signals shift multivesicular body (MVB) fate away from degradation and toward plasma membrane fusion, increasing EV release.

At the trafficking level, hypoxia-responsive signaling converges on Rab GTPase–dependent pathways that directly control EV secretion. Suppression of Rab7 limits lysosomal routes of MVBs, whereas upregulation of Rab27a and related Rab family members facilitates MVB docking and fusion with the plasma membrane, thereby amplifying EV output [42–44]. Ceramide-dependent membrane remodeling further supports vesicle budding and release under lipotoxic and inflammatory conditions prevalent in obesity [45]. Importantly, these mechanisms do not merely increase EV quantity but selectively bias EV cargo loading toward stress-adaptive and oncogenic programs.

**Fig. 2:**
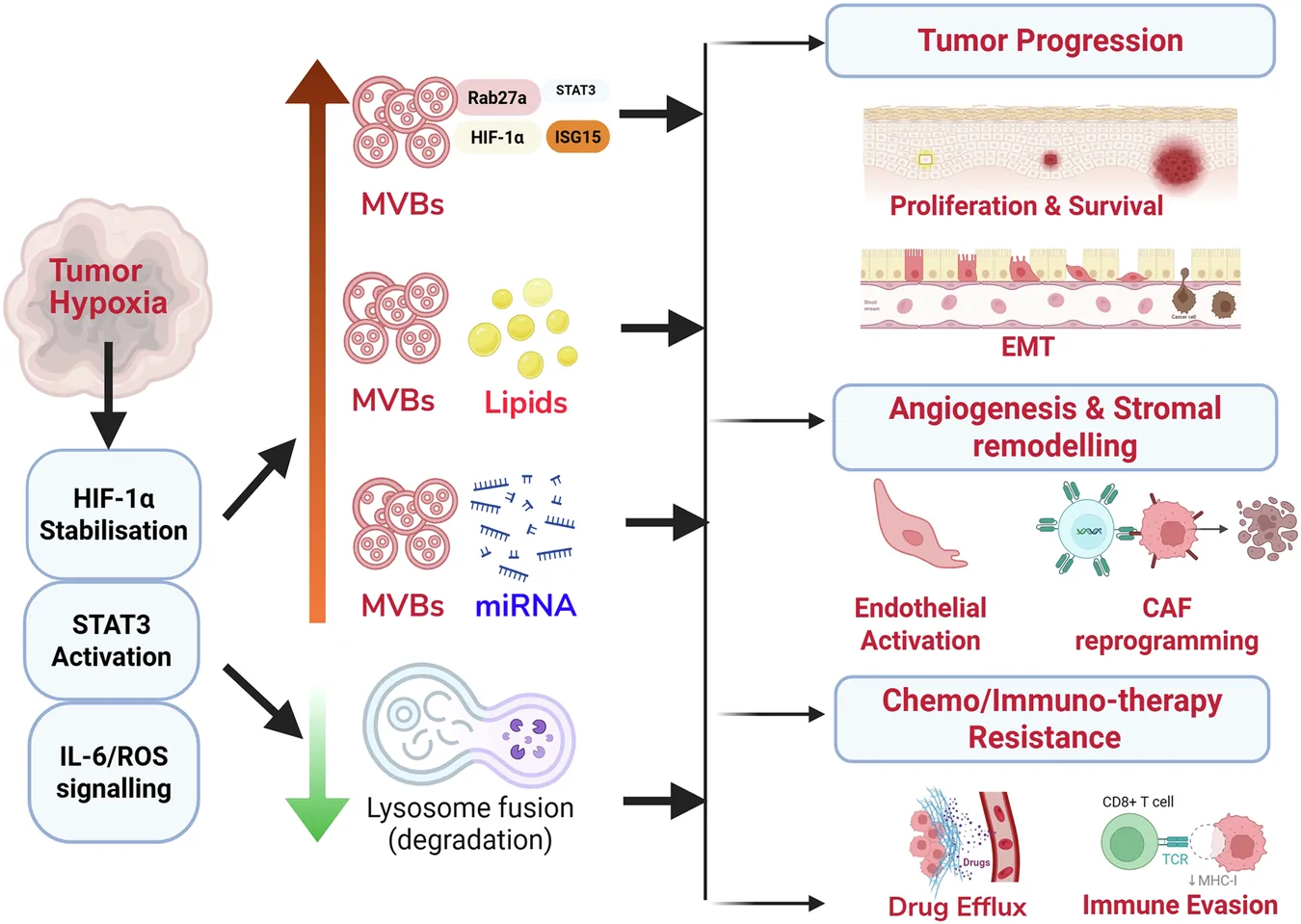
Obesity-associated hypoxia drives reprogramming of EV biogenesis and tumor-promoting signaling. Obesity-induced hypoxia activates HIF-1α and STAT3 signaling, shifting MVB trafficking toward Rab27a-mediated EV secretion. Hypoxia-conditioned EVs enriched with lipids, miRNAs, and stress proteins promote proliferation, EMT, angiogenesis, immune evasion, and therapy resistance.

Functionally, hypoxia-conditioned EVs are enriched in coordinated molecular modules, including bioactive lipids, regulatory RNAs, and stress-responsive proteins, that reinforce angiogenesis, epithelial plasticity, immune suppression, and therapy resistance in recipient cells [39, 46]. Thus, obesity-associated hypoxia acts not only as a tumor-promoting condition but as a systems-level regulator of EV-mediated intercellular communication, converting local adipose dysfunction into durable, long-range oncogenic signaling (Fig. 2).

## Extracellular vesicles versus soluble mediators in obesity-driven cancer signaling

Obesity-driven cancer progression is mediated by a complex network of soluble cytokines, adipokines, metabolites, immune-cell remodeling, and EV signaling [47, 48]. Rather than acting redundantly, these mediators play complementary and temporally distinct roles in shaping tumor ecosystems.

Soluble cytokines and adipokines, such as IL-6, TNF-α, leptin, and insulin, initiate and sustain inflammatory and growth-promoting signaling states but are inherently transient and subject to rapid dilution and degradation [49, 50]. In contrast, EVs encapsulate and protect bioactive cargo within lipid bilayers, enabling stable, long-distance transfer of multi-molecular programs derived from specific cellular sources. This compartmentalization allows EVs to deliver concentrated combinations of lipids, RNAs, and proteins that cooperatively reprogram recipient cells in ways not achievable by single soluble factors.

Conceptually, cytokines and adipokines often define the intensity of systemic signaling, whereas EVs define its content, persistence, and cellular specificity. EV uptake mechanisms can confer tissue and cell-type bias, enabling selective reprogramming of tumor cells, immune populations, stromal fibroblasts, and endothelial cells [32, 51]. This distinction helps explain why EV-associated signatures frequently outperform soluble biomarkers in discriminating disease states and why EVs emerge as particularly attractive substrates for multiplex biomarker development and mechanistic intervention in obesity-associated cancers.

## Streamlined oncogenic signaling pathways reinforced by obesity-conditioned EVs

Obesity-conditioned EVs reinforce tumor progression by converging on a limited set of conserved oncogenic signaling axes rather than activating disparate pathways independently. Among these, insulin/IGF-1–PI3K/AKT/mTOR, leptin–JAK/STAT3, ISG15, STAT5, TMEM205, Rab27a, and adiponectin–AMPK pathways represent dominant nodes through which metabolic dysfunction and EV signaling intersect [42, 52, 53].

Insulin resistance in obesity leads to compensatory hyperinsulinemia and increased bioavailability of insulin-like growth factor–1 (IGF-1), activating PI3K/AKT/mTOR and MAPK pathways that promote tumor cell proliferation, survival, metabolic adaptation, and resistance to apoptosis [54, 55]. EVs released under obese conditions amplify these signals by delivering regulatory RNAs and signaling proteins that sustain pathway activation in tumor and stromal cells, thereby reinforcing growth-promoting feedback loops.

Leptin-driven signaling constitutes a second major axis amplified by EV-mediated communication [56, 57]. Elevated leptin levels in obesity activate JAK2/STAT3, and STAT5 signaling, promoting angiogenesis, epithelial–mesenchymal transition (EMT), immune suppression, and metastatic potential [57, 58]. EV-associated STAT3-related cargo and leptin-responsive signaling components extend these effects beyond the immediate adipose environment, enabling coordinated activation of inflammatory and oncogenic programs within the tumor microenvironment [39, 59, 60].

In contrast, adiponectin signaling, normally protective through activation of AMP-activated protein kinase (AMPK), is suppressed in obesity. Reduced AMPK activity removes a critical inhibitory brake on inflammation, angiogenesis, and STAT3 signaling, further biasing EV-mediated communication toward tumor-promoting states [61]. In addition, we recently demonstrated that obesity-associated upregulation of TMEM205 and Rab27a enhances EV secretion and alters EV protein cargo, thereby promoting aggressive tumor progression and contributing to platinum resistance in ovarian and endometrial cancers [42, 53, 60, 62]. The net effect of these interactions is not pathway redundancy but pathway convergence, in which EVs function as integrative vectors that couple metabolic stress to oncogenic signaling across tissues.

Together, these streamlined signaling networks illustrate how obesity-conditioned EVs operate as central amplifiers of tumor-relevant pathways rather than as passive carriers of isolated molecular signals, reinforcing their mechanistic and translational significance.

## Extracellular vesicle–mediated remodeling of the tumor microenvironment

Obesity-driven EV signaling exerts its most profound oncogenic effects through coordinated remodeling of the tumor microenvironment (TME) [63–65]. Rather than acting on tumor cells in isolation, obesity-conditioned EVs reprogram immune, stromal, endothelial, and extracellular matrix compartments to establish a permissive niche for tumor growth, immune escape, and therapeutic resistance [66–69].

A dominant outcome of EV-mediated communication in obesity is suppression of anti-tumor immune surveillance. EVs enriched in inflammatory mediators, lipotoxic lipids, and regulatory RNAs promote the expansion and functional polarization of myeloid-derived suppressor cells (MDSCs), thereby impairing T-cell activation and cytotoxicity [70–72]. Concurrently, EV transfer of immunoregulatory proteins and microRNAs supports the recruitment and stabilization of regulatory T cells, reinforcing local immune tolerance [73, 74]. Obesity-associated EVs further compromise natural killer (NK) cell function by reducing activating receptor expression and disrupting cytolytic synapse formation, thereby weakening innate immune constraints on tumor progression [75, 76].

In parallel, EV signaling reprograms stromal fibroblasts toward cancer-associated fibroblast (CAF) phenotypes [77, 78]. EV-delivered oncogenic proteins and noncoding RNAs stimulate fibroblast proliferation, contractility, and secretion of pro-tumorigenic cytokines and growth factors [79]. Activated CAFs, in turn, reshape the vascular landscape by promoting endothelial activation and aberrant angiogenesis through EV-dependent delivery of angiogenic signals [80–82]. This disorganized neovasculature supports tumor expansion while simultaneously limiting effective immune cell infiltration and drug delivery.

**Fig. 3:**
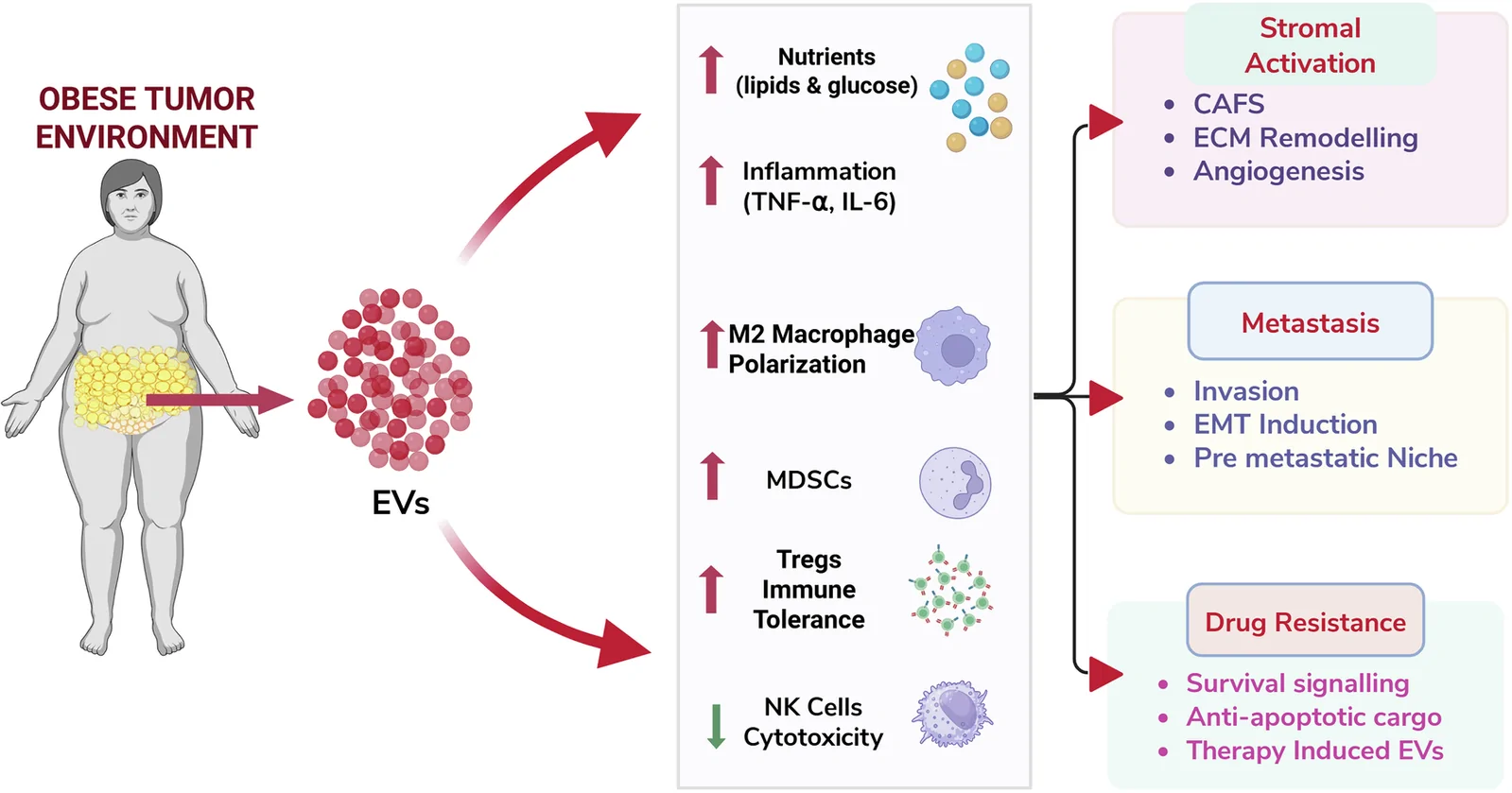
EV-mediated remodeling of the tumor microenvironment in obesity. Obesity-associated EVs remodel the tumor microenvironment by promoting immune suppression (M2 macrophages, MDSCs, Tregs; reduced NK activity) and activating stromal and endothelial compartments (CAFs, ECM remodeling, angiogenesis), driving invasion, metastasis, and treatment resistance.

Obesity-conditioned EVs also drive extracellular matrix (ECM) remodeling by inducing matrix-degrading enzymes and altering collagen organization, resulting in increased tissue stiffness and enhanced invasive potential [83]. EV-mediated activation of fibroblasts and stromal cells reinforces reciprocal signaling loops that sustain inflammation, angiogenesis, and immune suppression within the TME [32]. Collectively, these processes represent a coordinated EV-driven mechanism through which obesity transforms the TME from a reactive compartment into an active facilitator of malignant progression (Fig. 3).

## Shared versus cancer-specific EV mechanisms across obesity-associated malignancies

Although various solid tumors and metabolic cancers have been studied most extensively, emerging evidence indicates that EV-mediated mechanisms exhibit both shared and context-dependent features across obesity-associated malignancies [46, 84]. Common EV-driven outputs include immune suppression, stromal activation, epithelial plasticity, metabolic reprogramming, and angiogenesis, processes observed in breast, colorectal, pancreatic, hepatic, ovarian, and endometrial cancers [53, 85].

Cancer-type specificity arises from several factors. First, tissue-resident stromal and immune architectures influence EV uptake and downstream signaling responses [86]. Second, proximity to adipose depots and depot-specific adipose biology (omentum in ovarian cancer, visceral fat in colorectal and endometrial cancer) shapes EV exposure and organotropism [87]. Third, baseline oncogenic dependencies within tumor cells modulate sensitivity to EV-delivered cargo [32]. These context-dependent variables likely explain why similar EV programs can produce divergent phenotypic outcomes across tumor types.

Recognizing this balance between shared and cancer-specific EV mechanisms is essential for developing broadly applicable biomarker strategies while preserving tumor-type resolution in translational applications.

## Obesity-enhanced EV signaling in metastasis and pre-metastatic niche formation

Metastatic dissemination represents a major determinant of cancer mortality, and obesity markedly exacerbates metastatic risk across multiple malignancies [35, 88]. Obesity-conditioned EVs play a central role in this process by preparing distant tissues for tumor colonization prior to the arrival of circulating cancer cells [43, 89].

EVs derived from obese adipose tissue and tumor cells traffic to secondary organs, including liver, lung, omentum, ovarian and brain, where they remodel stromal, endothelial, and immune compartments [64, 69]. EV-delivered regulatory RNAs, inflammatory mediators, and metabolic enzymes induce vascular permeability, alter extracellular matrix composition, and suppress local immune surveillance, collectively creating permissive pre-metastatic niches [33, 42, 88].

Within target organs, EV signaling reprograms resident fibroblasts and immune cells toward tumor-supportive phenotypes while promoting endothelial activation and extravasation [82]. These changes lower the threshold for metastatic seeding and outgrowth, enabling disseminated tumor cells to survive and expand in otherwise restrictive microenvironments. Importantly, obesity increases both the abundance and pathogenic potency of circulating EVs, amplifying these pre-metastatic conditioning effects.

**Fig. 4:**
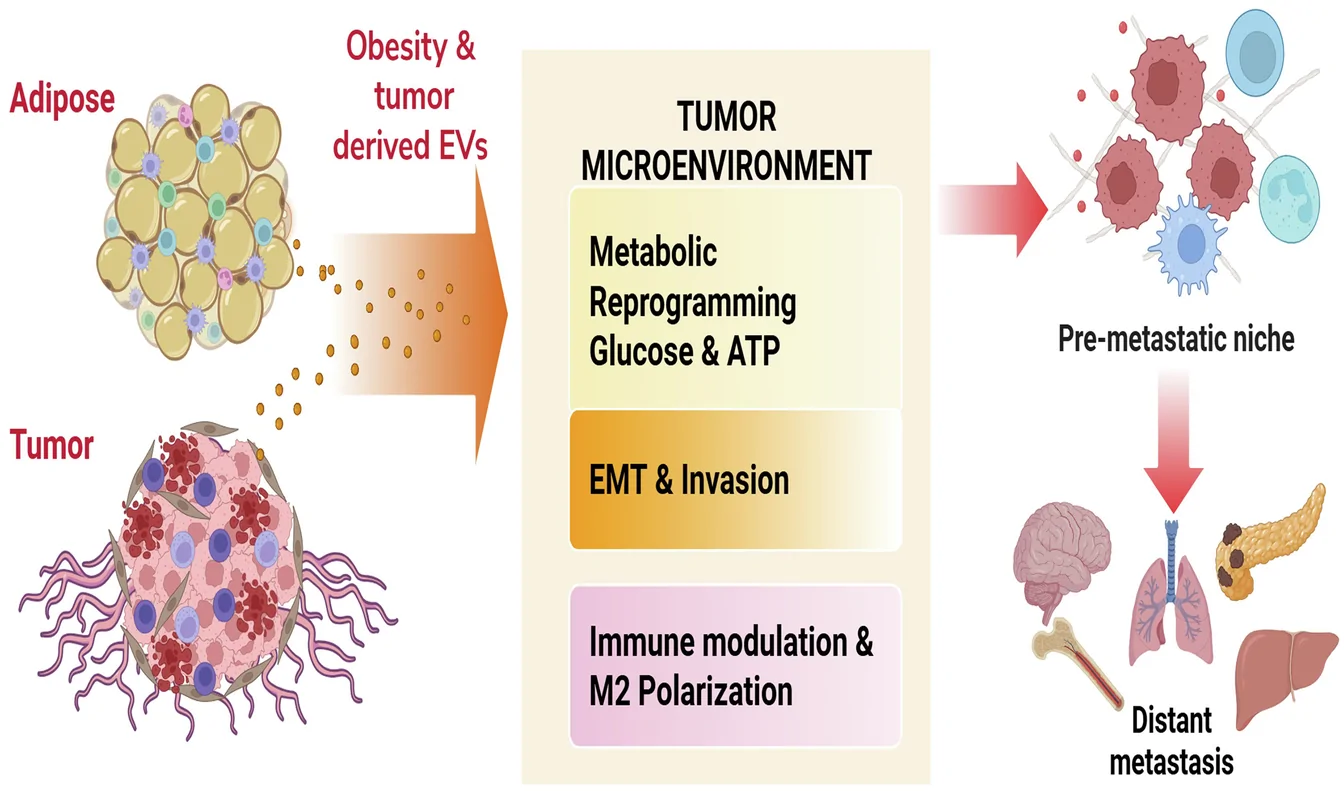
Obesity-enhanced EV signaling drives pre-metastatic niche formation and metastatic progression. EVs from obese adipose tissue and tumors condition distant organs by reprogramming stromal, endothelial, and immune cells, increasing vascular permeability and ECM remodeling to establish pre-metastatic niches that support tumor seeding and metastatic outgrowth.

Despite compelling evidence linking obesity-associated EVs to metastasis, key knowledge gaps remain regarding the relative contributions of adipocyte-, immune-, and tumor-derived EV populations, as well as the molecular determinants governing organ-specific EV uptake. Addressing these gaps will be critical for identifying therapeutic strategies to disrupt obesity-driven metastatic progression (Fig. 4).

## Clinical implications: extracellular vesicles as biomarkers and therapeutic targets

The growing burden of obesity-associated cancers has intensified the need for biomarkers capable of capturing early, mechanistically relevant tumor-associated changes through noninvasive sampling [35, 36, 90]. In this context, EVs offer distinct advantages over conventional circulating biomarkers. EVs are continuously released by adipocytes, immune cells, stromal cells, and tumor cells, and encapsulate coordinated molecular programs that reflect the physiological and pathological state of their cells of origin [91, 92]. Their membrane-bound structure protects cargo from degradation, enabling stable detection in biofluids such as plasma, urine, ascites, and saliva.

Accumulating translational evidence demonstrates that EV cargo profiles differ markedly between individuals with obesity-associated cancers and noncancer controls [35]. Disease-associated enrichment of EV proteins, DNAs and regulatory RNAs, including stress-responsive signaling mediators, metabolic enzymes, and oncogenic regulators, has been reported across multiple tumor types [93]. Importantly, several EV-associated alterations are detectable at early disease stages, supporting the potential utility of EVs for early detection, risk stratification, and longitudinal disease monitoring in cancers where delayed diagnosis remains a major driver of poor outcomes [35, 36, 53, 73].

Compared with traditional serum markers, EV-based assays offer improved analytical specificity by enriching tumor- and tissue-relevant signals while minimizing interference from abundant soluble plasma components [35]. Multiplex EV profiling further enables integration of protein, DNA, RNA, and lipid information within a single assay framework, enhancing discriminatory power and biological interpretability [94, 95]. Together, these features position EVs as a promising platform for biomarker development in obesity-associated malignancies.

**Fig. 5:**
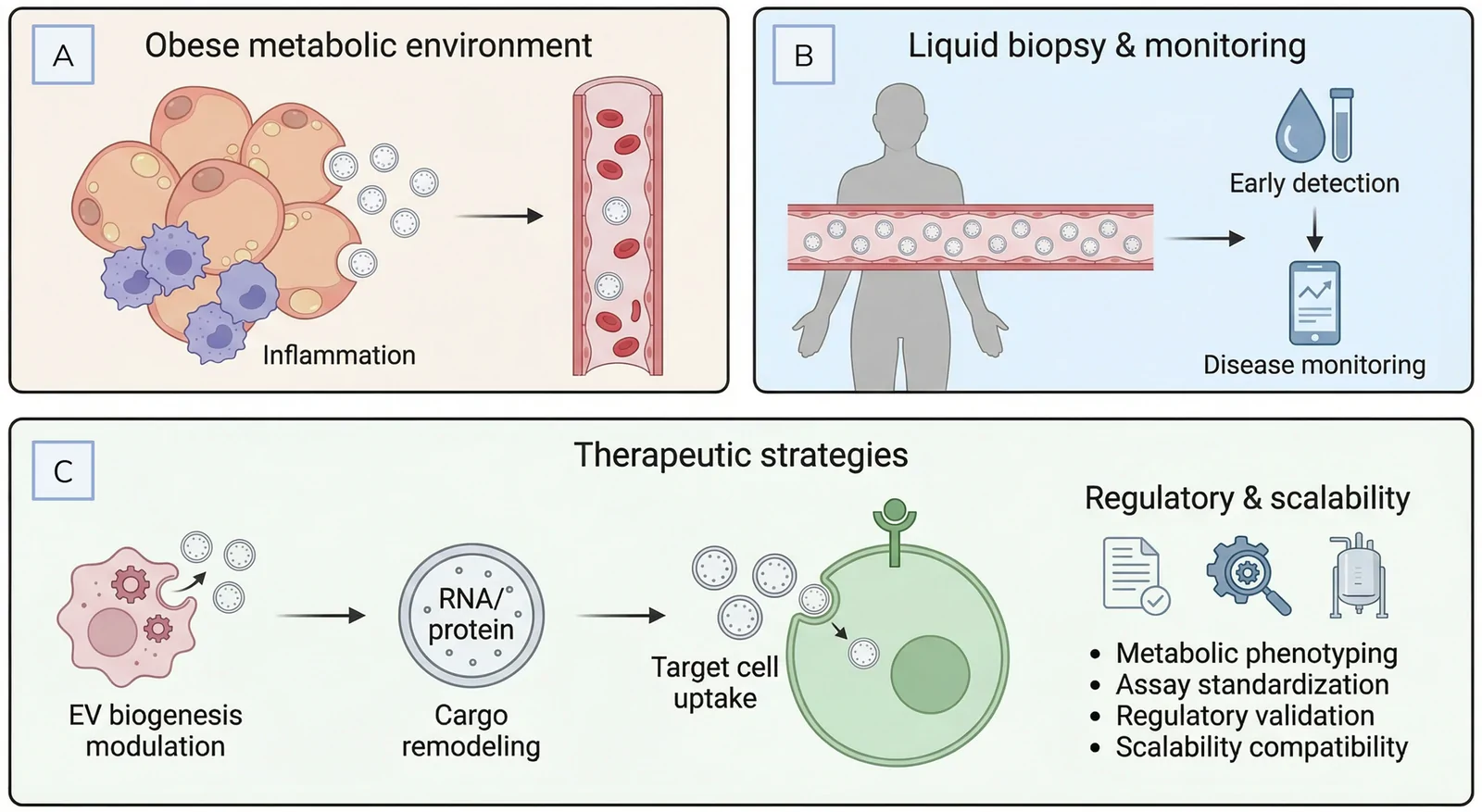
Clinical implications of EVs as biomarkers and therapeutic targets in obesity-associated cancers. **A** Obesity-driven metabolic inflammation increases systemic release of EVs from adipose, immune, stromal, and tumor cells. **B** Circulating EVs serve as stable, noninvasive biomarkers detectable in biofluids, enabling early cancer detection and longitudinal disease monitoring through liquid biopsy approaches. **C** Therapeutically, EV pathways can be targeted at multiple levels, including modulation of EV biogenesis, cargo remodeling, and inhibition of EV uptake, to disrupt tumor–host communication. Integration of EV-based diagnostics and therapeutics requires standardized assays, regulatory validation, and scalable platforms to enable clinical translation.

Beyond diagnostics, EVs represent actionable therapeutic targets. Obesity alters EV biogenesis, cargo loading, and uptake, amplifying tumor–host communication that promotes disease progression and treatment resistance [96]. Preclinical studies demonstrate that pharmacologic modulation of metabolic pathways, inhibition of EV release, blockade of EV uptake, and therapeutic engineering of EVs can attenuate tumor growth and metastatic potential [97]. Integrating EV-targeted strategies with metabolic and weight-loss interventions offers a rational approach to disrupt obesity-driven oncogenic signaling at its systemic source (Fig. 5).

## Inter-individual variability, scalability, and regulatory considerations

Successful clinical translation of EV-based diagnostics and therapeutics requires careful consideration of inter-individual variability and implementation challenges [12, 98]. EV abundance, cellular origin, and cargo composition are influenced by sex, age, ethnicity, body composition, metabolic health, menopausal status, medication exposures (including metabolic and anti-cancer therapies), and disease stage [96, 99]. These factors must be systematically incorporated into study design and analytical frameworks to ensure generalizability and clinical relevance.

Equally important are technical and regulatory considerations. Pre-analytical variables, including sample collection, processing time, storage conditions, and isolation methods, can substantially affect EV measurements [100, 101]. Standardized workflows for EV isolation, normalization, and reporting are essential to ensure reproducibility across studies and institutions. From a regulatory perspective, clinically deployable EV assays must demonstrate analytical validity, sensitivity, specificity, and robustness, with clear performance metrics and scalability compatible with routine clinical use. Addressing these challenges is critical for advancing EV-based tools from discovery to clinical implementation.

## Conclusions

Collectively, the evidence synthesized in this review identifies obesity-driven extracellular vesicle signaling as a unifying mechanism linking excess adiposity to cancer initiation, progression, immune evasion, metastasis, and therapy resistance. Obesity represents a chronic state of dysregulated intercellular communication in which EVs act as active vectors that disseminate coordinated inflammatory, metabolic, and oncogenic programs across tissues. By integrating adipose hypoxia, chronic inflammation, endocrine disruption, and altered vesicular trafficking, obesity reprograms EV biogenesis, cargo loading, and systemic distribution.

The convergence of metabolic signaling pathways, hypoxia-responsive transcriptional programs, and EV regulatory machinery position EVs as central effectors, rather than passive byproducts, of obesity-associated cancer biology. Importantly, the stability and molecular richness of EVs support strong potential for noninvasive detection, risk stratification, and longitudinal monitoring, while their biogenesis and uptake pathways offer tractable targets for therapeutic intervention.

## Future directions

Despite rapid progress, several key priorities must be addressed to translate obesity-driven EV biology into precision oncology. Mechanistic studies must move beyond descriptive cargo profiling toward causal interrogation of EV signaling axes in defined metabolic contexts. Functional perturbation strategies targeting EV biogenesis, cargo loading, and uptake tested in metabolically characterized models will be essential to determine when EVs act as dominant drivers versus amplifiers of obesity-associated tumor phenotypes.

High-resolution technologies, including single-EV profiling and spatially resolved analyses, are needed to dissect EV heterogeneity and define depot- and cell-of-origin–specific EV populations within adipose and tumor niches. Integration of these approaches with multi-omics EV profiling will enable the construction of mechanistically coherent EV signatures rather than static cargo lists.

Clinically, large, well-annotated longitudinal cohorts are required to move beyond BMI-based classification toward metabolically defined obesity phenotypes incorporating body composition, insulin resistance, inflammatory status, and treatment exposures. Harmonized analytical pipelines and reporting standards will be critical for reproducibility and cross-cohort validation. Finally, translational studies should test whether targeting EV-mediated inter-organ communication, through pharmacologic, metabolic, or lifestyle interventions, can reverse obesity-driven tumor progression or enhance therapeutic responsiveness. Together, these efforts will enable biomarker-guided surveillance and mechanism-based intervention in obesity-associated cancers.
